# KLRK1 as a prognostic biomarker for lung adenocarcinoma cancer

**DOI:** 10.1038/s41598-022-05997-z

**Published:** 2022-02-07

**Authors:** Yanan Zhang, Zeyang Chen, Aifang Jiang, Guanqi Gao

**Affiliations:** 1 Clinical Medical College, Weifang Medical University, Weifang, 261000 China; 2 Linyi People’s Hospital, Linyi, 276000 China; 3 Clinical Medical College, Qingdao University, Qingdao, 266000 China; 4 Weifang Medical University, Weifang, 261000 China

**Keywords:** Cancer, Lung cancer

## Abstract

Lung cancer is one of the most common malignancy worldwide and causes estimated 1.6 million deaths each year. Cancer immunosurveillance has been found to play an important role in lung cancer and may be related with its prognosis. KLRK1, encoding NKG2D, is a homodimeric lectin-like receptor. However, there has not been one research of KLRK1 as a biomarker in lung cancer. Data including patients` clinical characteristics and RNAseq information of KLRK1 from TCGA were downloaded. A total of 1019 patients with lung cancer were included in this study, among which 407 patients were female and 611 patients were male. Evaluations of mRNA expression, diagnostic value by ROC (receiver operating characteristic) curves and prognostic value by survival curve, Cox model and subgroup analysis were performed. The level of KLRK1 expression in lung adenocarcinoma cancer tissues and normal lung tissues was detected by qRT-PCR. The CCK-8 assay investigated the proliferation rate and the wound healing assay assessed the migratory ability in vitro. The expression of KLRK1 in tumor was lower than that in normal tissue. KLRK1 expression was associated with gender, histologic grade, stage, T classification and vital status. Patients with high KLRK1 expression presented an improved overall survival (P = 0.0036) and relapse free survival (P = 0.0031). KLRK1 was found to have significant prognostic value in lung adenocarcinoma (P = 0.015), stage I/II (P = 0.03), older patients (P = 0.0052), and male (P = 0.0047) by subgroup overall survival analysis, and in lung adenocarcinoma (P = 0.0094), stage I/II (P = 0.0076), older patients (P = 0.0072), and male (P = 0.0033) by subgroup relapse free survival analysis. Lung adenocarcinoma cancer patients with high KLRK1 expression presented an improved overall survival (P = 0.015) and relapse free survival (P = 0.0094). In vitro studies indicated that KLRK1 inhibited tumor cell proliferation and migration. KLRK1 was an independent prognostic factor and high KLRK1 expression indicated a better overall and relapse free survival. KLRK1 may be a prognostic biomarker for lung adenocarcinoma cancer.

## Introduction

Lung cancer is one of the most common malignancy worldwide and causes estimated 1.6 million deaths each year^1,2^. Depending on stage and regional differences, the 5-year survival rate of lung cancer varied from 4 to 17%^3^. Lung cancer is classified into non-small cell lung cancer (NSCLC) and small cell lung cancer (SCLC). SCLC only accounts for 15% of lung cancer cases, while the rest approximately 85% are NSCLC, among which lung adenocarcinoma and lung squamous cell carcinoma are the most common histological types^1,2^.

Cancer immunosurveillance has been found to play an important role in lung cancer and may be related with its prognosis. Recently, the role of KLRK1 in cancer immunosurveillance and immune escape is widely studied^4–6^. KLRK1, encoding NKG2D, is a homodimeric lectin-like receptor^7^. As a cytotoxic and co-stimulatory molecule on NK cells and T cells, KLRK1 is unique because it does not have any inhibitory isoforms^8,9^. KLRK1 has two kinds of ligands, which belong to MIC and RAET1 gene family^10^. The ligands of KLRK1 are frequently expressed on primary tumor cells and KLRK1 was reported to play a role in the control of tumor and infection^4,9^. Some studies have demonstrated the antitumor function of KLRK1, while some researchers argue that KLRK1 contributes to tumor growth in a model of inflammation-driven liver cancer^11,12^. As Neal et al. reviewed about developing biomarker-specific end points in lung cancer clinical trials, the exploration of biomarkers for a better diagnosis and prognosis is of great importance^13^. However, there has not been one research of KLRK1 as a biomarker in lung cancer.

In this study, expression of KLRK1 in patients with lung cancer was first evaluated. First, the relationship of KLRK1 and clinical features was examined. Then, the receiver operating characteristic (ROC) curves was plotted for analyzing the diagnostic value of KLRK1. To study the prognostic value of KLRK1, the survival package in R was used and Cox model was established, followed by evaluation of subgroups including different genders, different ages, and different stages.

## Methods

### Data mining

To collect original data from patients suffering from lung cancer, data mining was carried out. Specifically, we download data including patients’ clinical characteristics and RNAseq information of KLRK1 from TCGA (The Cancer Genome Atlas) database by UCSC Xena. Given that all the data were open to public, no ethical approval was needed. A total of 1019 patients with lung cancer were included in this study, among which 407 patients were female and 611 patients were male.

The RNA expression of KLRK1 was shown in boxplots as center line represents median, top line and bottom line of the box represents upper and lower quartiles and vertical lines represents 95% confidence intervals (95% CI).

To study the diagnostic value of KLRK1 in patients with lung cancer, ROC (Receiver operating characteristic) curves was plotted by pROC package^14^. The calculated area under curves (AUC) indicated the diagnostic value. Furthermore, according to the identified threshold level of KLRK1, the patients were grouped into high expression group and low expression.

To study the prognostic value of KLRK1 in patients with lung cancer, the survival package in R was used and Cox model was established^15^. Evaluation of subgroups was performed as well.

### Wound healing assay

Wound-healing assays were performed as previously described^16^. The migration of cells toward the wound was photographed under a Nikon fluorescence microscope.

### Immunohistochemical staining in HPA database and qRT-PCR

The Human Protein Atlas (HPA, http://www.proteinatlas.org/) online database was explored to validate the KLRK1 protein expression in lung cancer by immunohistochemical (IHC) staining by CAB021896 antibody^17^. Ten pairs of lung adenocarcinoma cancer tissues and normal lung tissues were obtained from primary adenocarcinoma cancer patients at the Affiliated Hospital of Weifang Medical University. According to the manufacturer’s instructions, total RNA was extracted using TRIzol reagent, cDNA was synthesized and the qRT-PCR was performed and calculated by means of 2−ΔΔCt methods. The related primer of KLRK1 were displayed as following: F: 5ʹ-TGGATTCGTGGTCGGAGGTCTC-3ʹ, R: 5ʹ-GGACATCTTTGCTTTTGCCATCGTG-3ʹ.

### Cell culture and cell transfection

A549 cell lines were purchased from American Tissue Culture Collection, and cultured in Dulbecco’s modified Eagle’s medium supplemented with 10% fetal bovine serum at 37 °C with 5% CO_2_.

The KLRK1 sequence was amplified and inserted into pCMV vector (Beyotime). The transfection of KLRK1 over-expression or control plasmid was performed using Lipofectamine 3000 (Invitrogen).

### Cell proliferation assay

The cells were treated with plasmids and cultured for 24 h. Then, 10 μL of CCK-8 reagent was added and cultured for 20 min. A microplate reader was used to measure the absorbance at 490 nm. The cell viability was calculated relative to the untreated control.

To evaluate the cell viability, co-staining of calcein AM and PI was performed. The cells were seeded and incubated for 16 h. After different treatments for 24 h, the cells were stained and observed using a Nikon fluorescence microscope.

### Wound healing assay

Wound-healing assays were performed as previously described^16^. The migration of cells toward the wound was photographed under a Nikon fluorescence microscope.

### Statistical analysis

R version 3.5.2 packages (https://www.R-project.org) was used for bioinformatics analysis^18^. Data were presented using the ggplot2 package in R^19^. The Wilcoxon rank-sum test was used for comparison between two groups, and the Kruskal–Wallis test was used for comparison among three or more groups. For assessment of associations between KLRK1 expression and clinical parameters, the chi-squared test was used, and corrected by Fisher’s exact test. Data from in vitro and in vivo experiments were analyzed by the Student’s t-test (unpaired, two-tailed). P < 0.05 was statistical significance.

### Ethical approval

This study was approved by the ethics committee of the Affiliated Hospital of Weifang Medical University and conducted in strict accordance with the National Institutes of Health guidelines.

## Results

### Characteristics of patients with lung cancer

Clinical characteristics of the patients with lung cancer, including age, gender, histological type, stage, T classification, N classification, M classification, radiation therapy, residual tumor, vital status, sample type, KLRK1 expression, were shown in Table 1. The percentage of two histological types, lung adenocarcinoma (50.74%) and lung squamous cell carcinoma (49.26%), was close. About half of the patients were in stage I (51.13%). As for T, N, M classification, T2 (56.04%), N0 (63.98%), and M0 (74.39%) were highest among each classification. Most patients (99.80%) were primary tumor.

**Table 1:** Clinical characteristics of the patients with lung cancer.

Characteristics	Number (%)
**Age**	
< 55	109 (10.70)
≥ 55	881 (86.46)
NA	29 (2.84)
**Gender**	
Female	407 (39.94)
Male	611 (59.96)
NA	1 (0.10)
**Histological type**	
Lung adenocarcinoma	517 (50.74)
Lung squamous cell carcinoma	502 (49.26)
**Stage**	
I	521 (51.13)
II	284 (27.87)
III	168 (16.49)
IV	33 (3.24)
NA	13 (1.28)
**T classification**	
T1	284 (27.87)
T2	571 (56.04)
T3	118 (11.58)
T4	42 (4.12)
TX	3 (0.29)
NA	1 (0.10)
**N classification**	
N0	652 (63.98)
N1	227 (22.28)
N2	114 (11.19)
N3	7 (0.69)
NX	17 (1.67)
NA	2 (0.20)
**M classification**	
M0	758 (74.39)
M1	32 (3.14)
MX	220 (21.59)
NA	9 (0.88)
**Radiation therapy**	
NO	782 (76.74)
YES	110 (10.79)
NA	127 (12.46)
**Residual tumor**	
R0	743 (72.91)
R1	25 (2.45)
R2	8 (0.79)
RX	48 (4.71)
NA	195 (19.14)
**Vital status**	
Deceased	404 (39.65)
Living	614 (60.26)
NA	1 (0.10)
**Sample type**	
Primary tumor	1017 (99.80)
Recurrent tumor	2 (0.20)
**KLRK1 expression**	
High	701 (68.79)
Low	318 (31.21)

*NA* not available, *X* represents uncertain.

### Low KLRK1 expression in tumor

**Figure 1:**
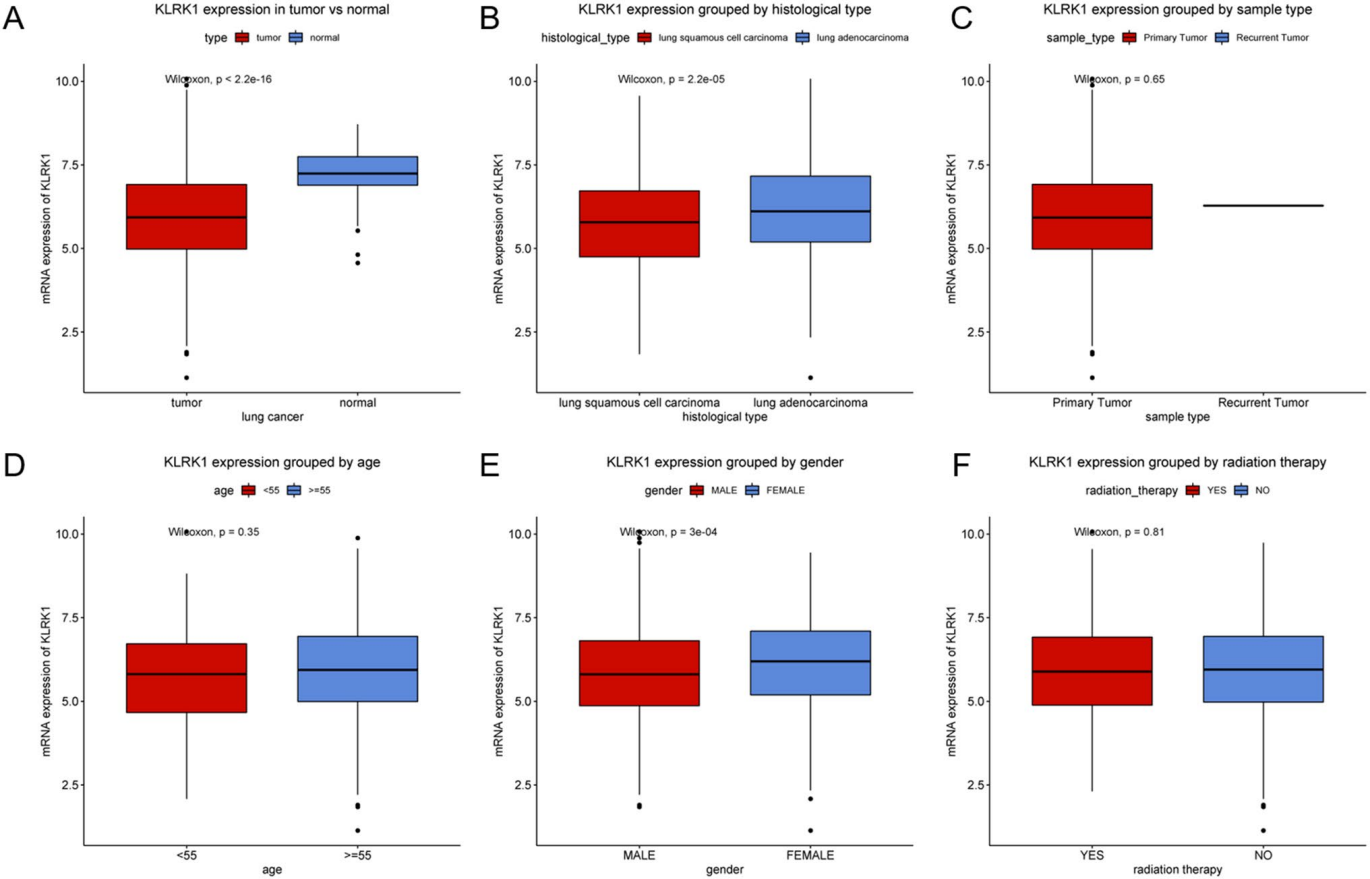
KLRK1 expression in lung cancer. Expression of KLRK1 grouped by (**A**) tumor vs. normal tissue, (**B**) histological type, (**C**) sample type, (**D**) age, (**E**) gender, and (**F**) radiation therapy.

As shown in Fig. 1, the expression of KLRK1 in tumor was lower than that in normal tissue (P < 2.2e−16). The association of KLRK1 expression with clinical features, including histological type, sample type, age, gender, radiation therapy, was further evaluated. KLRK1 expression in lung squamous cell carcinoma was lower than that in lung adenocarcinoma (P = 2.2e−05). KLRK1 expression in male was lower than that in female (P = 3e−04). Other clinical features did not show statistical differences (P > 0.05).

**Figure 2:**
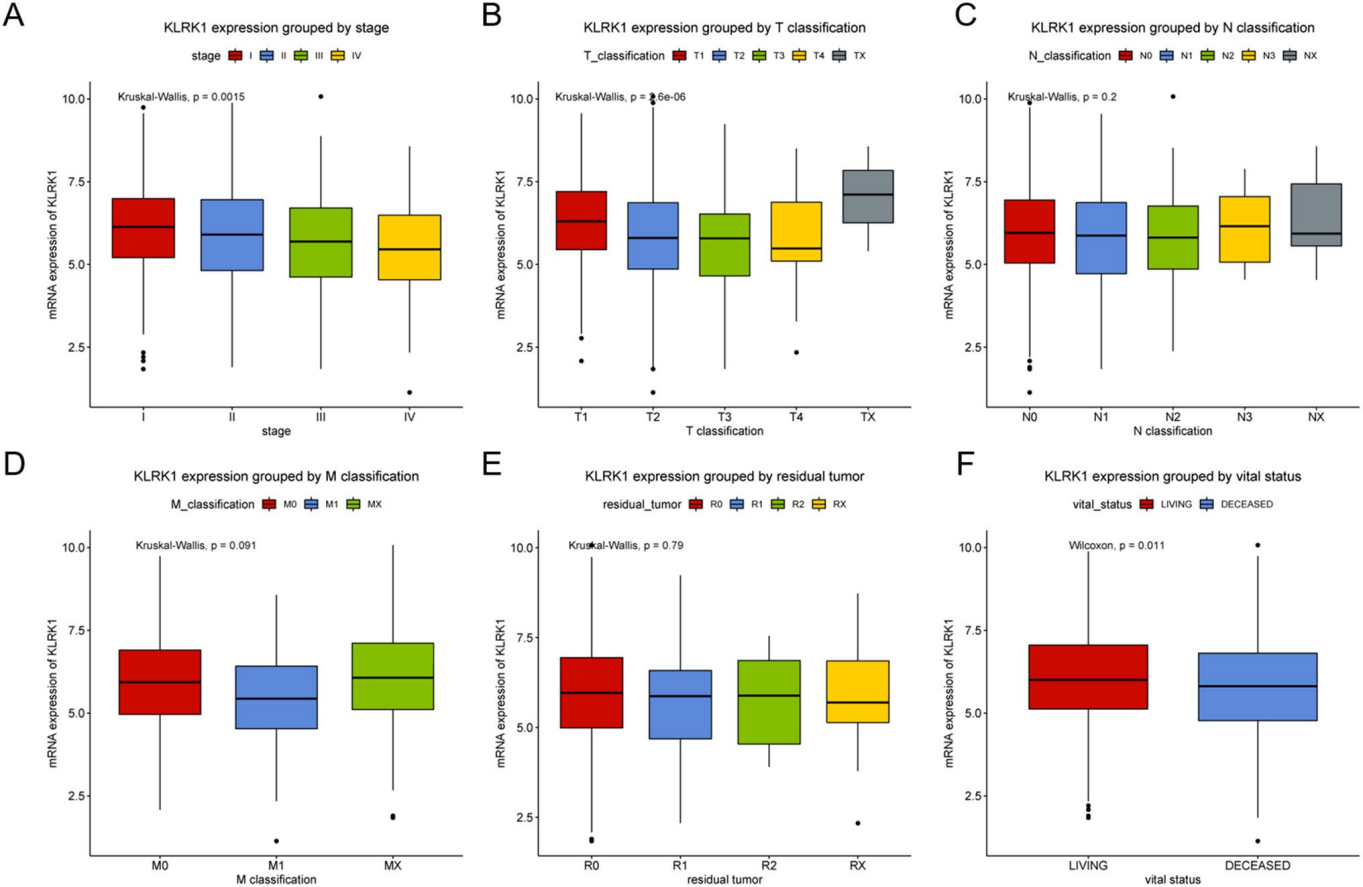
KLRK1 expression in lung cancer. Expression of KLRK1 grouped by (**A**) stage, (**B**) T classification, (**C**) N classification, (**D**) M classification, (**E**) residual tumor and (**F**) vital status.

Association of KLRK1 expression with stage, T classification, N classification, M classification, residual tumor and vital status was evaluated (Fig. 2). The KLRK1 expression got decreased progressively with higher stages (P = 0.0015) and T classification (P = 2.6e−06). No significances were observed in N classification (P = 0.200), M classification (P = 0.091) and residual tumor (P = 0.790). The KLRK1 expression in living patients was a little higher than that in deceased patients (P = 0.011).

### Diagnostic value of KLRK1 for lung cancer

**Figure 3:**
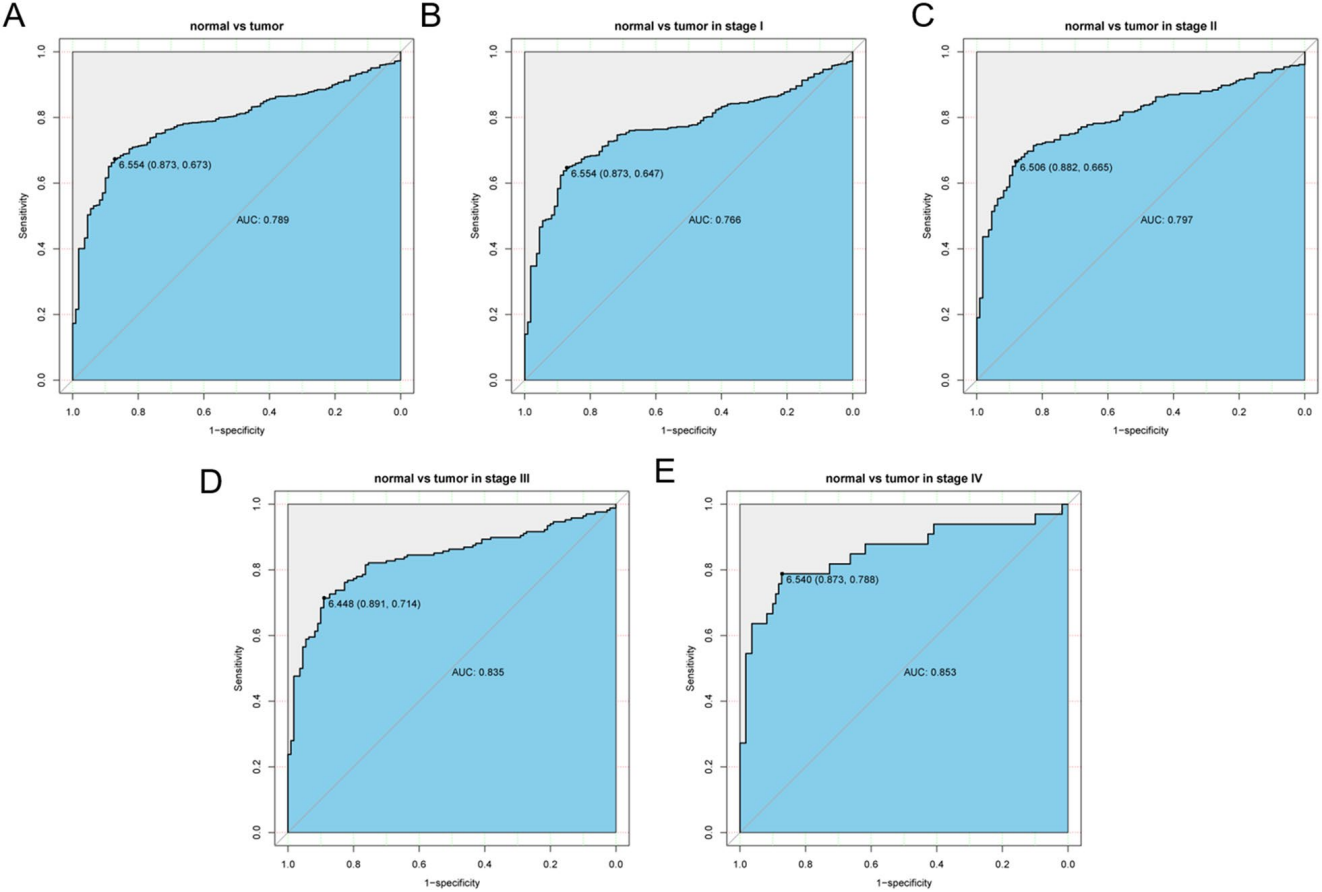
ROC (Receiver operating characteristic) curves of KLRK1 expression in patients with lung cancer. (**A**) Normal vs. overall, (**B**) stage I, (**C**) stage II, (**D**) stage III and (**E**) stage IV tumor.

As shown in Fig. 2, the ROC analysis was first performed in all lung cancer patients, indicating a modest diagnostic value with AUC of 0.789. Moreover, different stages of lung cancer were analyzed. It was suggested that the diagnostic value was increasing with stage getting higher from stage I (AUC = 0.766) to stage II (AUC = 0.797) to stage III (AUC = 0.835) finally to stage IV (AUC = 0.853) (Fig. 3).

### Correlation of KLRK1 expression with clinical features

According to the threshold value ascertained by ROC analysis, patients were divided to two subgroups, high (n = 701) and low (n = 318) KLRK1 expression group. The relationship between the clinical features and KLRK1 expression in patients with lung cancer was shown in Table 2. KLRK1 expression was associated with gender (P = 0.005), histologic grade (P = 0.001), stage (P = 0.001), T classification (P < 0.001) and vital status (P = 0.001). No correlation was found between KLRK1 expression and age (P = 0.431), N classification (P = 0.218), M classification (P = 0.383), radiation therapy (P = 0.399), residual tumor (P = 0.336), and sample type (P = 0.850).

**Table 2:** Relationship between the clinical features and KLRK1 expression in patients with lung cancer.

Clinical characteristics	Variable	Number of patients	KLRK1				χ^2^	P value
			High	%	Low	%		
Age	< 55	109	71	(10.41)	38	(12.34)	0.620	0.431
	≥ 55	881	611	(89.59)	270	(87.66)		
Gender	Female	407	301	(42.94)	106	(33.44)	7.819	**0.005**
	Male	611	400	(57.06)	211	(66.56)		
Histological type	Lung adenocarcinoma	517	381	(54.35)	136	(42.77)	11.285	**0.001**
	Lung squamous cell carcinoma	502	320	(45.65)	182	(57.23)		
Stage	I	521	386	(55.86)	135	(42.86)	15.336	**0.001**
	II	284	181	(26.19)	103	(32.70)		
	III	168	102	(14.76)	66	(20.95)		
	IV	33	22	(3.18)	11	(3.49)		
T classification	T1	284	229	(32.67)	55	(17.35)	29.391	** < 0.001**
	T2	571	373	(53.21)	198	(62.46)		
	T3	118	69	(9.84)	49	(15.46)		
	T4	42	27	(3.85)	15	(4.73)		
	TX	3	3	(0.43)	0	(0)		
N classification	N0	652	461	(65.86)	191	(60.25)	5.610	0.218
	N1	227	146	(20.86)	81	(25.55)		
	N2	114	75	(10.71)	39	(12.30)		
	N3	7	4	(0.57)	3	(0.95)		
	NX	17	14	(2.00)	3	(0.95)		
M classification	M0	758	516	(74.03)	242	(77.32)	1.904	0.383
	M1	32	21	(3.01)	11	(3.51)		
	MX	220	160	(22.96)	60	(19.17)		
Radiation therapy	No	782	540	(88.38)	242	(86.12)	0.711	0.399
	Yes	110	71	(11.62)	39	(13.88)		
Residual tumor	R0	743	513	(90.80)	230	(88.8)	3.305	0.336
	R1	25	14	(2.48)	11	(4.25)		
	R2	8	4	(0.71)	4	(1.54)		
	RX	48	34	(6.02)	14	(5.41)		
Vital status	Deceased	404	253	(36.09)	151	(47.63)	11.673	**0.001**
	Living	614	448	(63.91)	166	(52.37)		
Sample type	Primary tumor	1017	699	(99.71)	318	(100)	0.036	0.850
	Recurrent tumor	2	2	(0.29)	0	(0)		

X represents uncertain.

### IHC and qRT-PCR result

**Figure 4:**
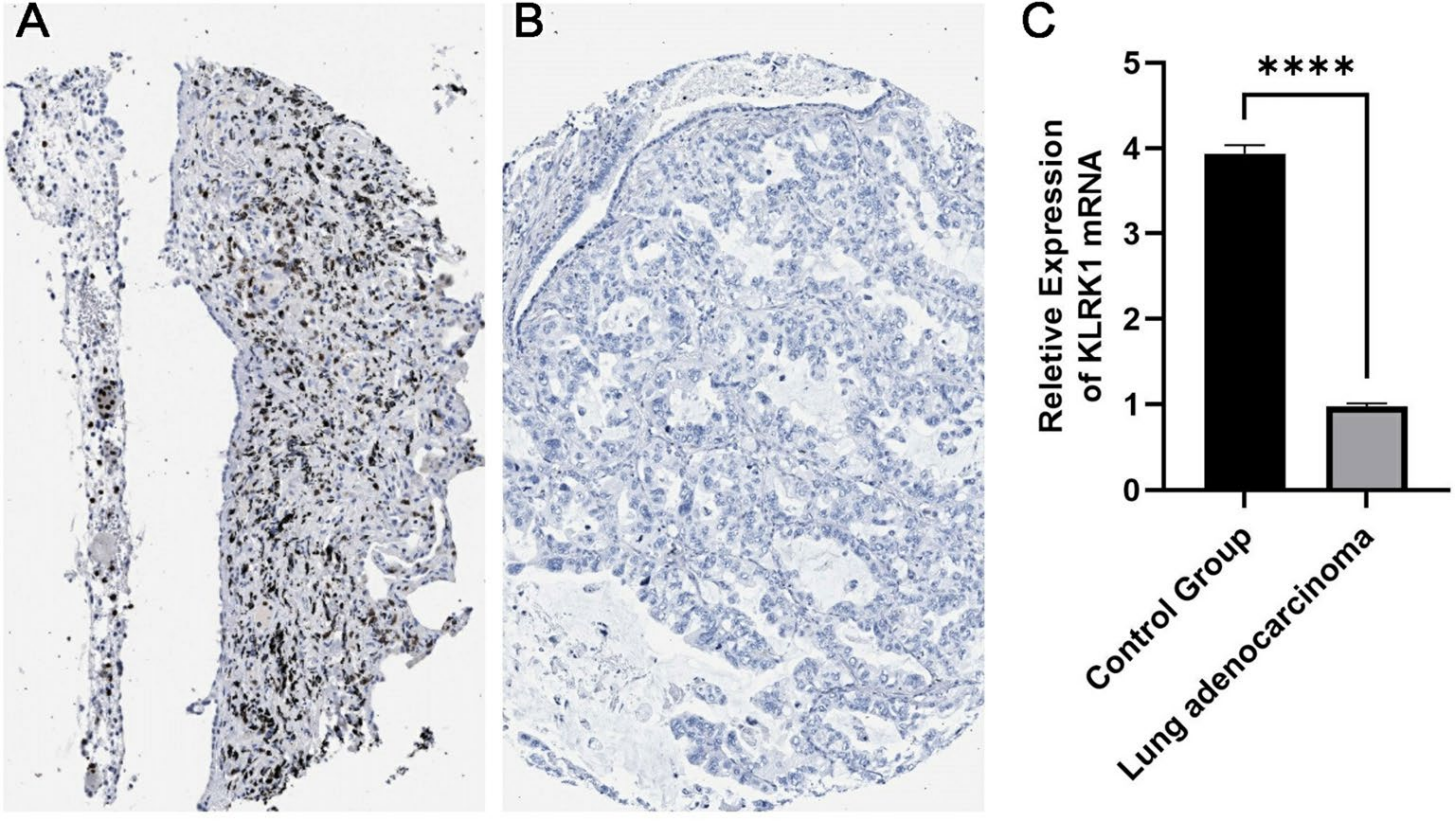
IHC of KLRK1 expression in normal lung tissue (**A**) and lung adenocarcinoma tissue (**B**), qRT-PCR of KLRK1 in two tissues (**C**).

Comparing the IHC experimental pictures obtained in the HPA database, it can be seen that the expression of KLRK1 in lung adenocarcinoma cancer tissues was significantly lower than that in normal lung tissues. As shown in Fig. 4, we utilized qRT-PCR to validate the KLRK1 expression in lung adenocarcinoma cancer tissues and found the KLRK1 expression was down-regulated in the lung adenocarcinoma cancer (N = 10) compared with normal lung tissues (N = 10; P < 0.001).

### Overall survival and relapse free survival of KLRK1 for lung cancer

**Figure 5:**
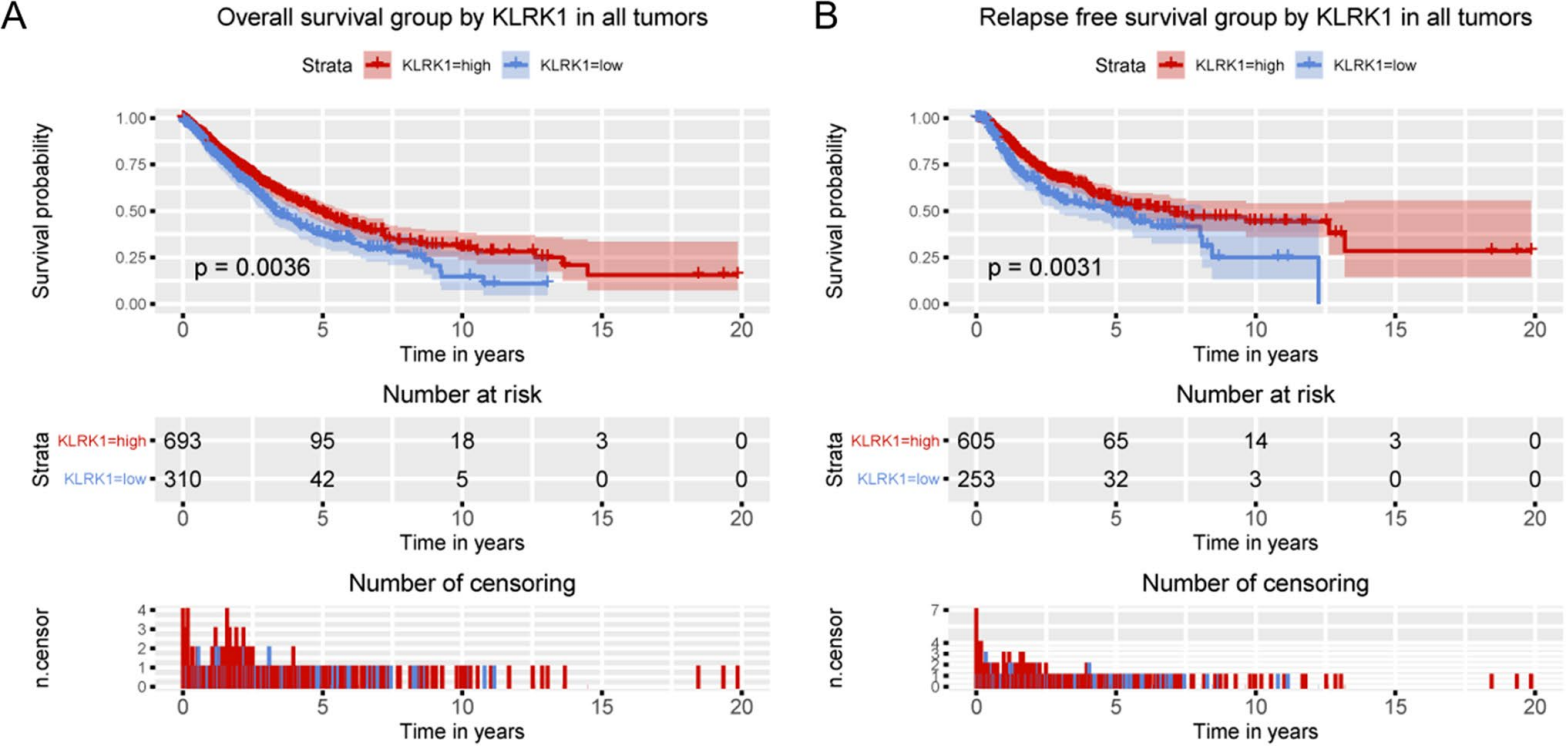
Relationship of KLRK1 expression with (**A**) overall and (**B**) relapse free survival in all patients with lung cancer.

**Figure 6:**
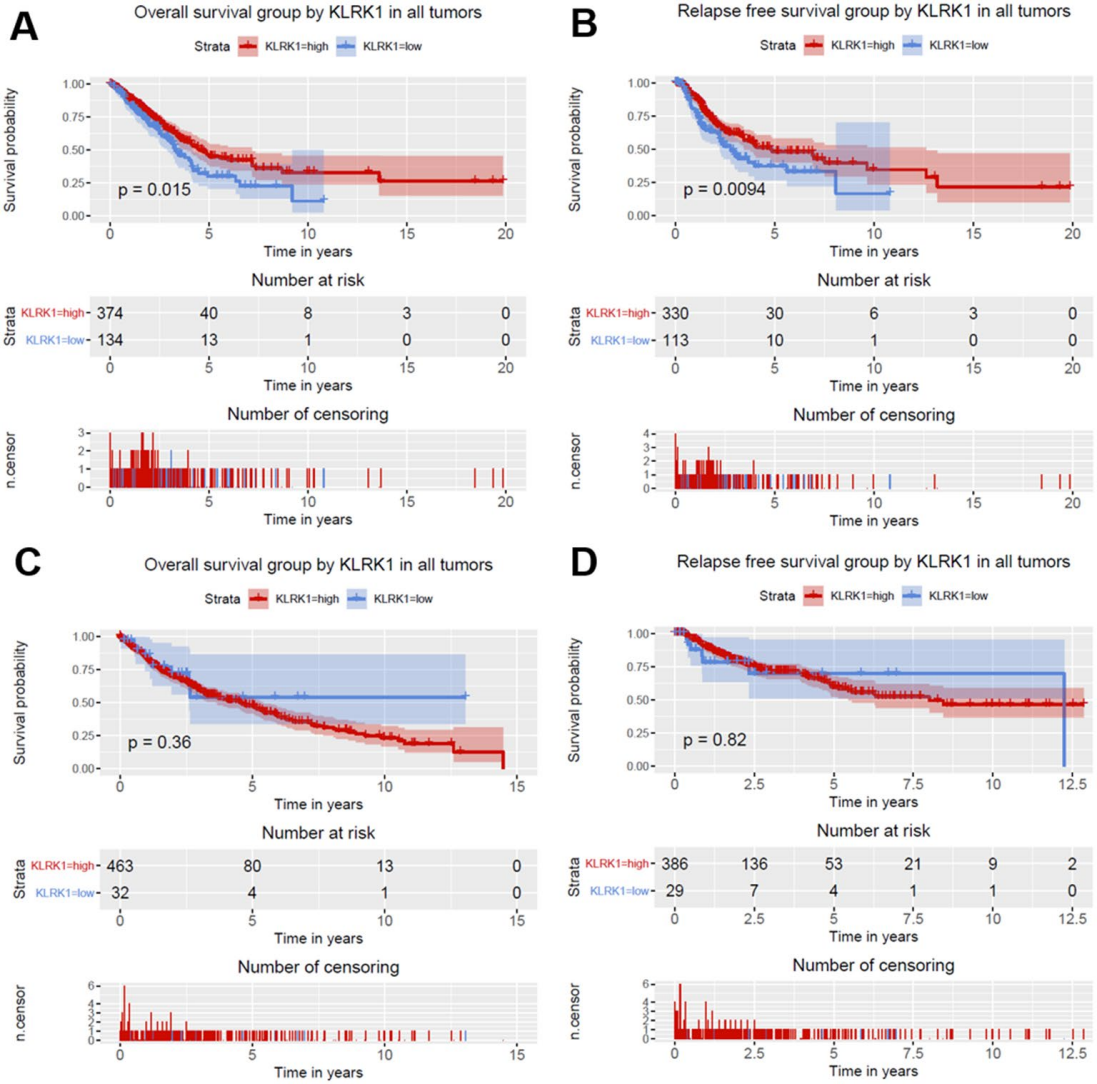
Relationship of KLRK1 expression with (**A**) overall and (**B**) relapse free survival in patients with lung adenocarcinoma cancer, and (**C**) overall and (**D**) relapse free survival in patients with lung squamous cell cancer.

Given that KLRK1 was correlated with survival, the prognostic value of KLRK1 was further studied. As shown in Fig. 5, lung cancer patients with high KLRK1 expression presented an improved overall survival (P = 0.0036) and relapse free survival (P = 0.0031). As shown in Fig. 6, lung adenocarcinoma cancer patients with high KLRK1 expression presented an improved overall survival (P = 0.015) and relapse free survival (P = 0.0094). But no significances were found in lung squamous cell cancer patients with high KLRK1 expression.

### Subgroup analysis of KLRK1 for lung cancer’s overall survival

**Figure 7:**
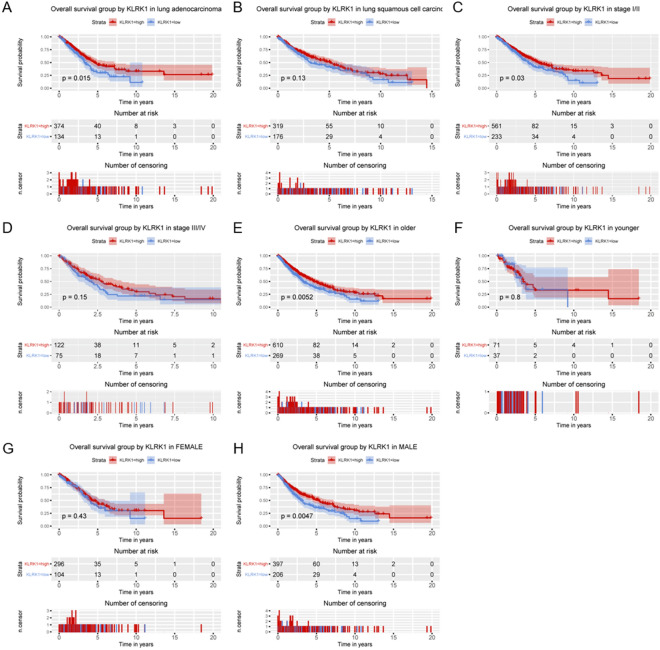
Relationship of KLRK1 expression with overall survival in all patients with lung cancer in (**A**) lung adenocarcinoma, (**B**) lung squamous cell carcinoma, (**C**) stage I/II, (**D**) stage III/IV, (**E**) older patients, (**F**) younger patients, (**G**) female and (**H**) male.

By overall survival analysis of subgroups (Fig. 7, Figs. S1, S2), KLRK1 was found to have significant prognostic value in lung adenocarcinoma (P = 0.015), stage I/II (P = 0.03), older patients (P = 0.0052), and male (P = 0.0047). For lung adenocarcinoma cancer, KLRK1 was found to have significant prognostic value in stage I/II (P = 0.049), older patients (P = 0.0064), and male (P = 0.013).

### Subgroup analysis of KLRK1 for lung cancer’s relapse free survival

**Figure 8:**
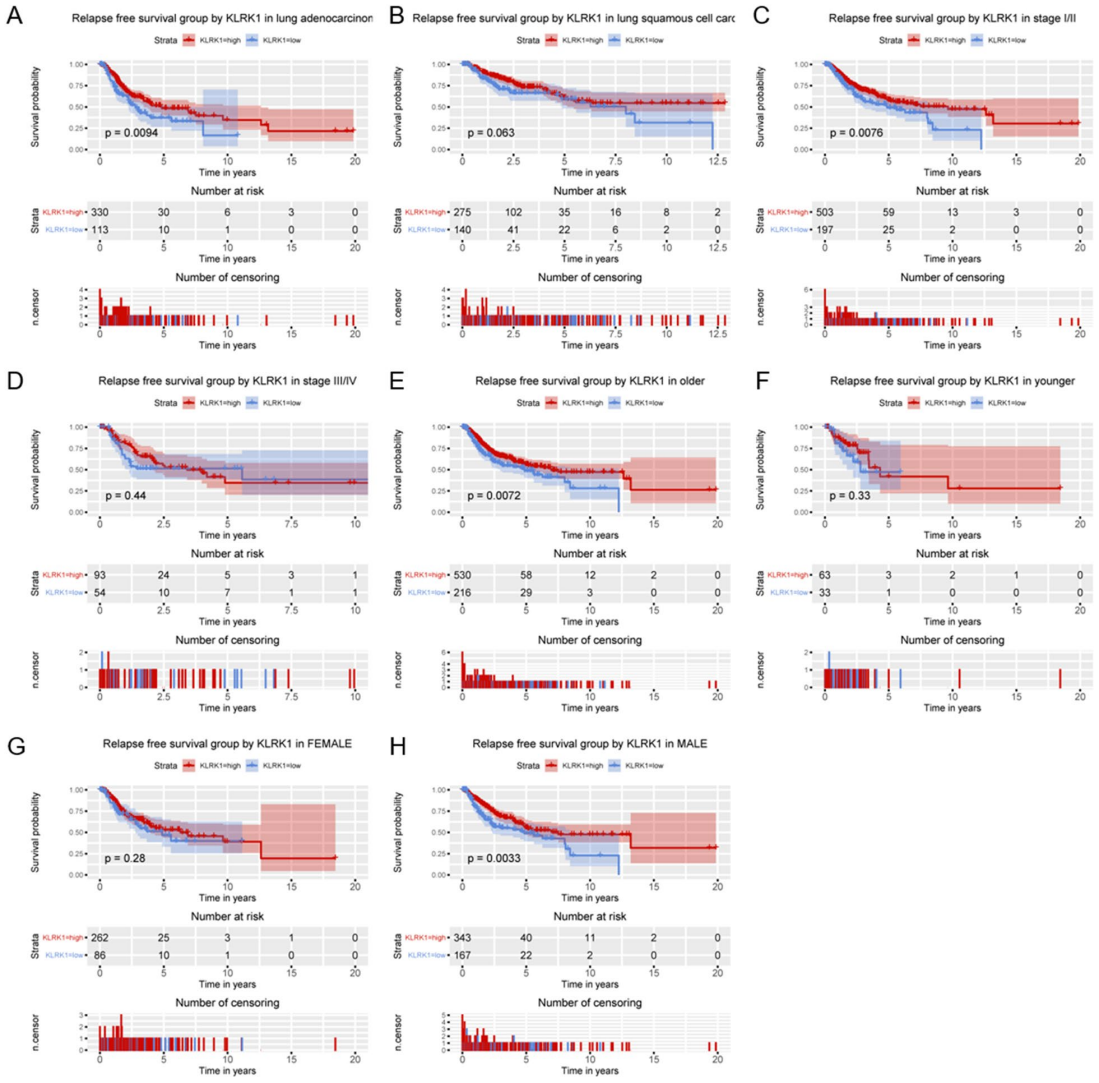
Relationship of KLRK1 expression with relapse free survival in all patients with lung cancer in (**A**) lung adenocarcinoma, (**B**) lung squamous cell carcinoma, (**C**) stage I/II, (**D**) stage III/IV, (**E**) older patients, (**F**) younger patients, (**G**) female and (**H**) male.

By relapse free survival analysis of subgroups (Fig. 8, Figs. S3, S4), KLRK1 was found to have significant prognostic value in lung adenocarcinoma (P = 0.0094), stage I/II (P = 0.0076), older patients (P = 0.0072), and male (P = 0.0033). For lung adenocarcinoma cancer, KLRK1 was found to have significant prognostic value in stage I/II (P = 0.0025), and older patients (P = 0.012).

### KLRK1 is an independent risk factor for lung cancer’s overall survival

The univariate Cox model of overall and relapse free survival in patients with lung cancer was established (Tables 3, 4, Table S1), and the multivariate analysis of overall and relapse free survival in patients with lung cancer was further performed (Tables 5, 6, Table S2).

**Table 3:** Univariate analysis of overall and relapse free survival in patients with lung cancer.

	Overall survival			Relapse free survival		
	Hazard ratio	95% CI	P value	Hazard ratio	95% CI	P value
Age	1.03	0.74–1.43	0.867	0.95	0.63–1.43	0.819
Gender	1.14	0.93–1.40	0.201	0.94	0.73–1.20	0.613
Histological type	1.12	0.91–1.36	0.282	0.63	0.49–0.82	** < 0.001**
T classification	1.42	1.25–1.61	** < 0.001**	1.35	1.14–1.59	** < 0.001**
N classification	1.39	1.22–1.57	** < 0.001**	1.41	1.20–1.65	** < 0.001**
M classification	1.16	0.91–1.49	0.220	0.83	0.62–1.11	0.210
Radiation therapy	1.00	0.79–1.27	0.983	1.76	1.32–2.35	** < 0.001**
Residual tumor	1.17	1.03–1.33	**0.016**	1.29	1.11–1.49	**0.001**
Stage	1.48	1.33–1.64	** < 0.001**	1.41	1.23–1.62	** < 0.001**
KLRK1 expression	0.74	0.60–0.91	**0.004**	0.68	0.52–0.88	**0.003**

**Table 4:** Univariate analysis of overall and relapse free survival in patients with lung adenocarcinoma cancer.

	Overall survival			Relapse free survival		
	Hazard ratio	95% CI	P value	Hazard ratio	95% CI	P value
Age	0.82	0.55–1.22	0.322	–	–	–
Gender	1.05	0.78–1.4	0.760	–	–	–
T classification	1.52	1.26–1.82	** < 0.001**	1.16	0.96–1.4	0.132
N classification	1.67	1.41–1.97	** < 0.001**	1.15	0.92–1.44	0.228
M classification	1.4	1.04–1.9	**0.029**	0.93	0.68–1.28	0.662
Radiation therapy	1.25	0.89–1.75	0.199	–	–	–
Residual tumor	1.2	1.01–1.42	**0.037**	1.14	0.96–1.35	0.150
Stage	1.68	1.47–1.93	** < 0.001**	1.51	1.21–1.89	** < 0.001**
KLRK1	0.69	0.51–0.93	**0.016**	0.78	0.57–1.07	0.129

**Table 5:** Multivariate analysis of overall survival and relapse free survival in patients with lung cancer.

	Overall survival			Relapse-free survival		
	Hazard ratio	95% CI	P value	Hazard ratio	95% CI	P value
Histological type	–	–	–	0.58	0.45–0.76	** < 0.001**
T classification	1.18	1.02–1.36	**0.025**	1.22	1.01–1.47	**0.040**
N classification	–	–	–	1.11	0.89–1.4	0.343
Radiation therapy	–	–	–	1.42	1.06–1.91	**0.018**
Residual tumor	1.13	0.99–1.28	0.067	1.23	1.07–1.42	**0.004**
Stage	1.34	1.14–1.57	** < 0.001**	1.16	0.93–1.43	0.183
KLRK1	0.79	0.64–0.97	**0.025**	0.74	0.57–0.96	**0.022**

**Table 6:** Multivariate analysis of overall survival and relapse free survival in patients with lung adenocarcinoma cancer.

	Overall survival			Relapse free survival		
	Hazard ratio	95% CI	P value	Hazard ratio	95% CI	P value
T classification	1.39	1.13–1.71	**0.002**	1.18	0.94–1.49	0.155
N classification	1.45	1.2–1.76	** < 0.001**	1.12	0.85–1.48	0.410
Radiation therapy	1.97	1.37–2.82	** < 0.001**	1.62	1.12–2.35	**0.010**
Residual tumor	1.20	1.01–1.43	**0.035**	1.16	0.98–1.38	0.088
Stage	1.44	1.23–1.7	** < 0.001**	1.17	0.91–1.51	0.209
KLRK1	0.64	0.45–0.9	**0.010**	0.74	0.52–1.06	0.099

As shown in Table 3, no obvious differences were observed in age (P = 0.867), gender (P = 0.201), histological type (P = 0.282), M classification (P = 0.220), and radiation therapy (P = 0.983). T classification (P < 0.001), N classification (P < 0.001), residual tumor (P = 0.016), stage (P < 0.001) and KLRK1 expression (P = 0.004) showed significant differences for lung cancer. As shown in Table 4, T classification (P < 0.001), N classification (P < 0.001), M classification (P = 0.029), residual tumor (P = 0.037), stage (P < 0.001) and KLRK1 expression (P = 0.016) showed significant differences for lung adenocarcinoma cancer. As shown in Table 5, T classification (HR 1.18; 95% CI 1.02–1.36; P = 0.025) and stage (HR 1.34; 95% CI 1.14–1.57; P < 0.001) were risk factors for lung cancer.

### KLRK1 is an independent risk factor for lung cancer’s relapse free survival

As shown in Table 3, no obvious differences were observed in age (P = 0.819), gender (P = 0.613), and M classification (P = 0.210). Histological type (P < 0.001), T classification (P < 0.001), N classification (P < 0.001), radiation therapy (P < 0.001), residual tumor (0.001), stage (P < 0.001) and KLRK1 expression (P = 0.003) showed significant differences. As shown in Table 4, stage (P < 0.001) showed significant differences for lung adenocarcinoma cancer. As shown in Table 5, T classification (HR 1.22; 95% CI 1.01–1.47; P = 0.040), radiation therapy (HR 1.42; 95% CI 1.06–1.91; P = 0.018) and residual tumor (HR 1.23; 95% CI 1.07–1.42; P = 0.004) were risk factors for lung cancer.

### KLRK1 inhibits lung cancer cell proliferation and migration in vitro

**Figure 9:**
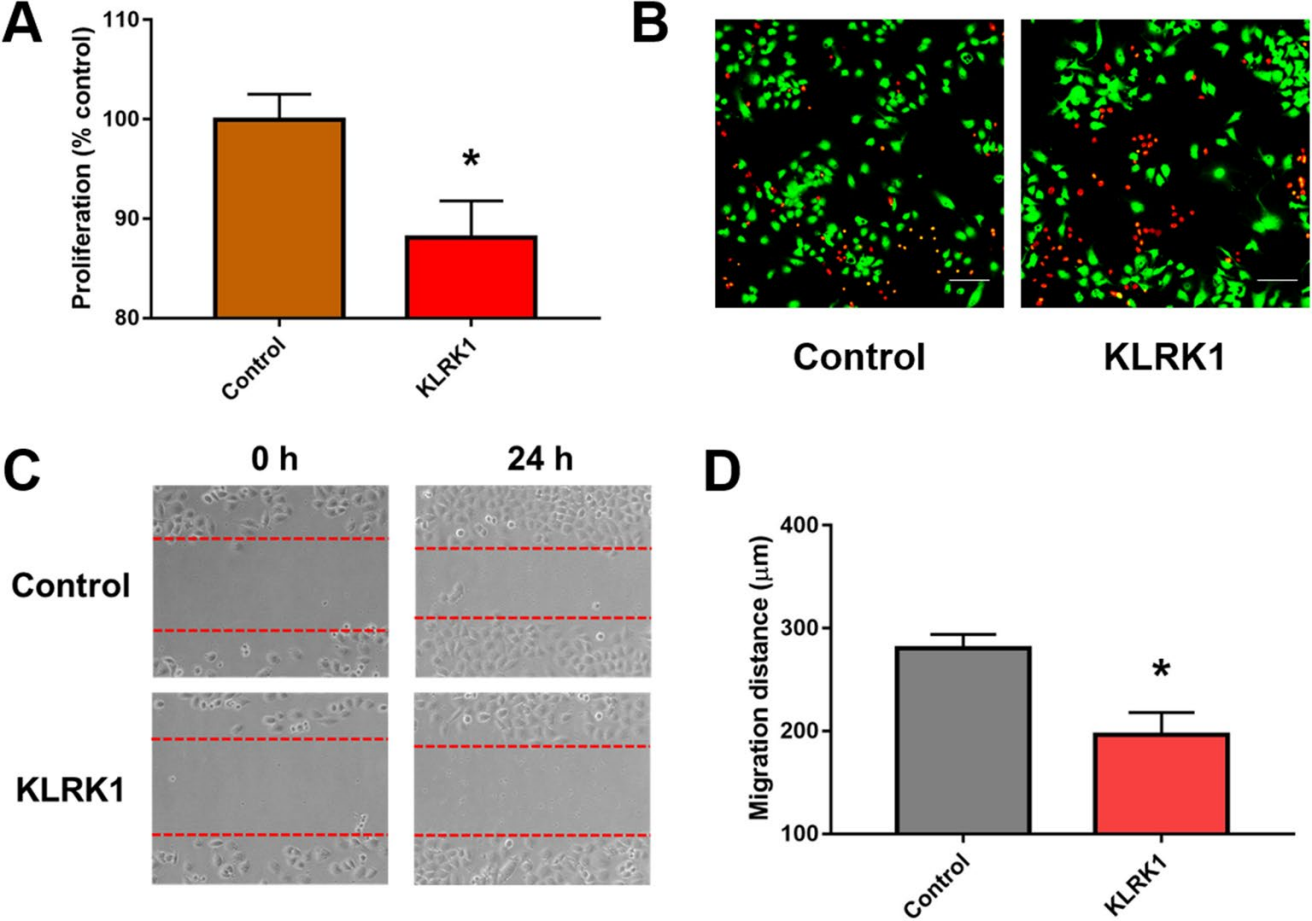
The effect of KLRK1 in lung cancer A549 cell lines. (**A**) The CCK-8 assay; (**B**) live/dead staining; (**C**) the migration and (**D**) migration distance.

We further studied the effect of KLRK1 on cells by overexpressing KLRK1 in lung cancer A549 cell lines. The CCK-8 assay showed that KLRK1 upregulation significantly inhibited cell proliferation (P < 0.05; Fig. 9A). Compared with control group, KLRK1 increased the percentage of dead cells by live/dead staining (Fig. 9B). KLRK1 decreased the migration distance of cancer cell (P < 0.05; Fig. 9C,D).

## Discussion

Lung cancer is one of the most common malignancy with poor prognosis worldwide. Many researches have working on mining novel prognostic biomarker in different types of cancer^20–36^. However, little is done in the field of lung cancer. In this bioinformatics study, KLRK1 was found to lower expressed in lung cancer compared with normal lung tissue. KLRK1 expression was associated with gender, histologic grade, stage, T classification and vital status. Moreover, KLRK1 presented a moderate diagnostic value for lung cancer.

Recently, research about biomarkers by data mining is popular^20,21,23,24,27,30,36–40^. Although some protein blood biomarkers have already been put into clinical practice, the ability of their diagnosis and prognosis is limited and he exploration of biomarkers for lung cancer is ongoing^13^. CEA (carcinoembryonic antigen) and CTC (Circulating tumor cells) are used in some lung cancers^41,42^. Also, there are studies reporting some novel biomarkers. Jiang et al. found thymidine kinase 1 combined with CEA, CYFRA21-1 and NSE improved its diagnostic value for lung cancer^43^. Yang et al. compared the different value of combination in CEA, CA125 (carbohydrate antigen 125), CY211 (cytokeratin 19 fragment), NSE (neuron-specific enolase), and SCC (squamous cell carcinoma antigen)^44^. Tang et al. identified HSP70 (circulating heat shock protein 70) as a novel biomarker for early diagnosis of lung cancer^45^. In this study, we first found KLRK1 as a prognostic biomarker for lung cancer.

Researches of biomarkers for lung cancer are not only limited in prognosis but also in diagnosis. Wu et al. found UCK2 (Uridine‐cytidine kinase 2) as a potential diagnostic and prognostic biomarker for lung cancer, and identified UCK2 highly expressed in stage IA lung cancer with AUC > 0.9^46^. Jiang et al. reported a diagnostic value of circulating lncRNA XLOC_009167 with AUC of 0.7398^47^. Similarly, we found the KLRK1 presented a promising diagnostic value in lung cancer, especially advanced stages. In comparison of lung cancer *vs.* tumor, the AUC could get into 0.789, indicating a relatively high diagnostic value of KLRK1 as a biomarker for lung cancer.

KLRK1 is an immunoreceptor binding to a variety of cell surface glycoproteins distantly related to MHC class I molecules^48^. A recent study conducted by Shi et al. reported the up-regulation of KLRK1-activating receptors that recognize lung cancer could facilitate the clearance of lung cancer^49^. Their results confirmed the role of KLRK1 in changing state of NK cells, leading to the control of lung cancer through immunosurveillance. Paczulla et al. reported the absence of KLRK1 ligand mediated the immune evasion of leukaemia stem cells^30^. Dong et al. found T cells expressing KLRK1 chimeric antigen receptors could efficiently eliminate glioblastoma and cancer stem cells^34^. Moreover, blockade drugs for EGFR and PD-1 can enhance the effective of NKG2D, encoded by KLRK1, lead to cancer cell recognition and killing by NK effector cells^50,51^. From our results, high KLRK1 expression is associated with a better overall and relapse free survival, which may attributes to the immunosurveillance of KLRK1 therefore suppressing the proliferation and metastasis of lung cancer^4^. Of note, our results showed the significances of KLRK1 in both overall survival and relapse free survival in lung cancer.

Our research first suggested the diagnostic and prognostic value of KLRK1 for lung cancer. However, the major limitation is that this study analyzed the data from a single database by data mining. Further verifications in different areas and populations are required. Besides, in vivo function experiments and exploration of its molecular mechanism would further illuminate the role of KLRK1 in lung cancer.

## Conclusions

In conclusion, KLRK1 was lower expressed in lung cancer in comparison with normal lung tissue. KLRK1 expression was associated with gender, histologic grade, stage, T classification and vital status. KLRK1 had a diagnostic value for lung adenocarcinoma cancer. KLRK1 was an independent prognostic factor and high KLRK1 expression indicated a better overall and relapse free survival. KLRK1 may be a novel biomarker for lung adenocarcinoma cancer.

## Supplementary Information

Supplementary Information.
