# Supplementary material for: KLRK1 as a prognostic biomarker for lung adenocarcinoma cancer
Source: Sci Rep. 2022 Feb 7;12:1976. doi: 10.1038/s41598-022-05997-z (PMC8821622; doi:10.1038/s41598-022-05997-z)
Supplement: Supplementary file 1 — Supplementary Information. [file 41598_2022_5997_MOESM1_ESM.docx]

**KLRK1 as a Prognostic Biomarker** **for Lung Adenocarcinoma Cancer**

Yanan Zhang^1,2^, Zeyang Chen^3,4^, Guanqi Gao^2※^

1 Clinical Medical College, Weifang Medical University,Weifang, 261000, China

2 Linyi People’s Hospital, Linyi, China, 276000

3 Clinical Medical College, Qingdao University, Yantai, China, 266000

4 The Affiliated Yantai Yuhuangding Hospital of Qingdao University, Yantai, China, 264000

^※^Corresponding. lygqgao@163.com

**Table S1.** Univariate analysis of overall and relapse free survival in patients with lung squamous cell cancer

|  | Overall survival | | | Relapse free survival | | |
| --- | --- | --- | --- | --- | --- | --- |
|  | Hazard Ratio | 95%CI | P value | Hazard Ratio | 95%CI | P value |
| Age | 1.59 | 0.84-3 | 0.155 | - | - | - |
| Gender | 1.20 | 0.87-1.65 | 0.273 | - | - | - |
| T classification | 1.34 | 1.12-1.6 | **0.001** | 1.25 | 1.01-1.55 | **0.044** |
| N classification | 1.12 | 0.93-1.36 | 0.239 | - | - | - |
| M classification | 0.77 | 0.52-1.15 | 0.206 | - | - | - |
| Radiation therapy | 0.84 | 0.6-1.17 | 0.295 | - | - | - |
| Residual tumor | 1.12 | 0.93-1.36 | 0.229 | - | - | - |
| Stage | 1.26 | 1.07-1.48 | **0.006** | 1.12 | 0.91-1.37 | 0.281 |
| KLRK1 | 1.36 | 0.7-2.66 | 0.365 | - | - | - |

**Table S2.** Multivariate analysis of overall and relapse free survival in patients with lung squamous cell cancer

|  | Overall survival | | | Relapse free survival | | |
| --- | --- | --- | --- | --- | --- | --- |
|  | Hazard Ratio | 95%CI | P value | Hazard Ratio | 95%CI | P value |
| T classification | 1.44 | 1.1-1.89 | **0.009** | 1.25 | 0.87-1.8 | 0.234 |
| N classification | 1.34 | 1.02-1.76 | **0.033** | 1.11 | 0.73-1.68 | 0.629 |
| Radiation therapy | 1.45 | 0.89-2.36 | 0.132 | - | - | - |
| Residual tumor | 1.49 | 1.16-1.91 | **0.002** | 1.46 | 1.13-1.88 | **0.004** |
| Stage | 1.38 | 1.09-1.76 | **0.008** | 1.18 | 0.76-1.83 | 0.465 |
| KLRK1 | 0.92 | 0.42-1.99 | 0.825 | - | - | - |


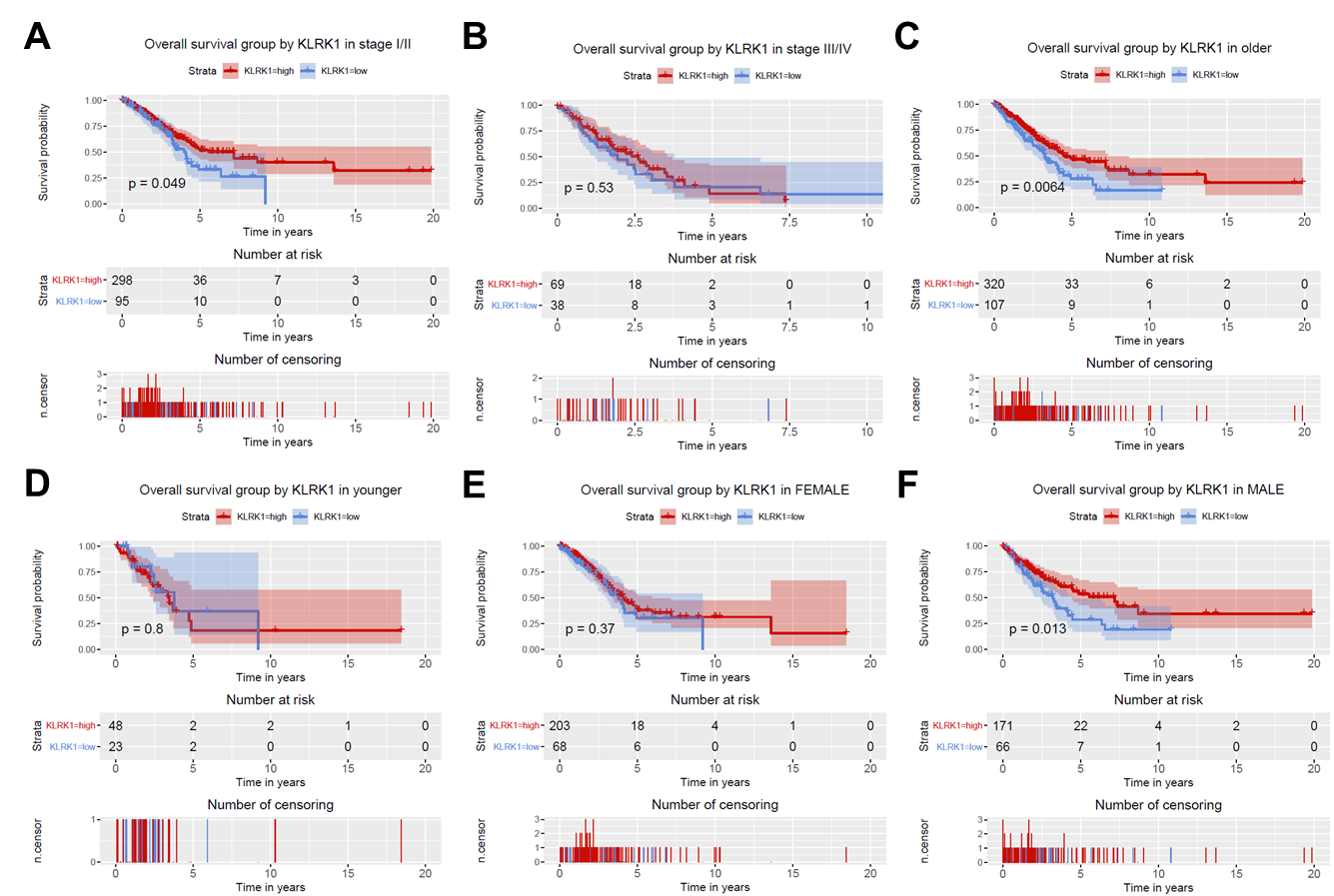


**Figure S1.** Relationship of KLRK1 expression with overall survival in all patients with lung adenocarcinoma cancer in (A) stage I/II, (B) stage III/IV, (C) older patients, (D) younger patients, (E) female and (F) male.


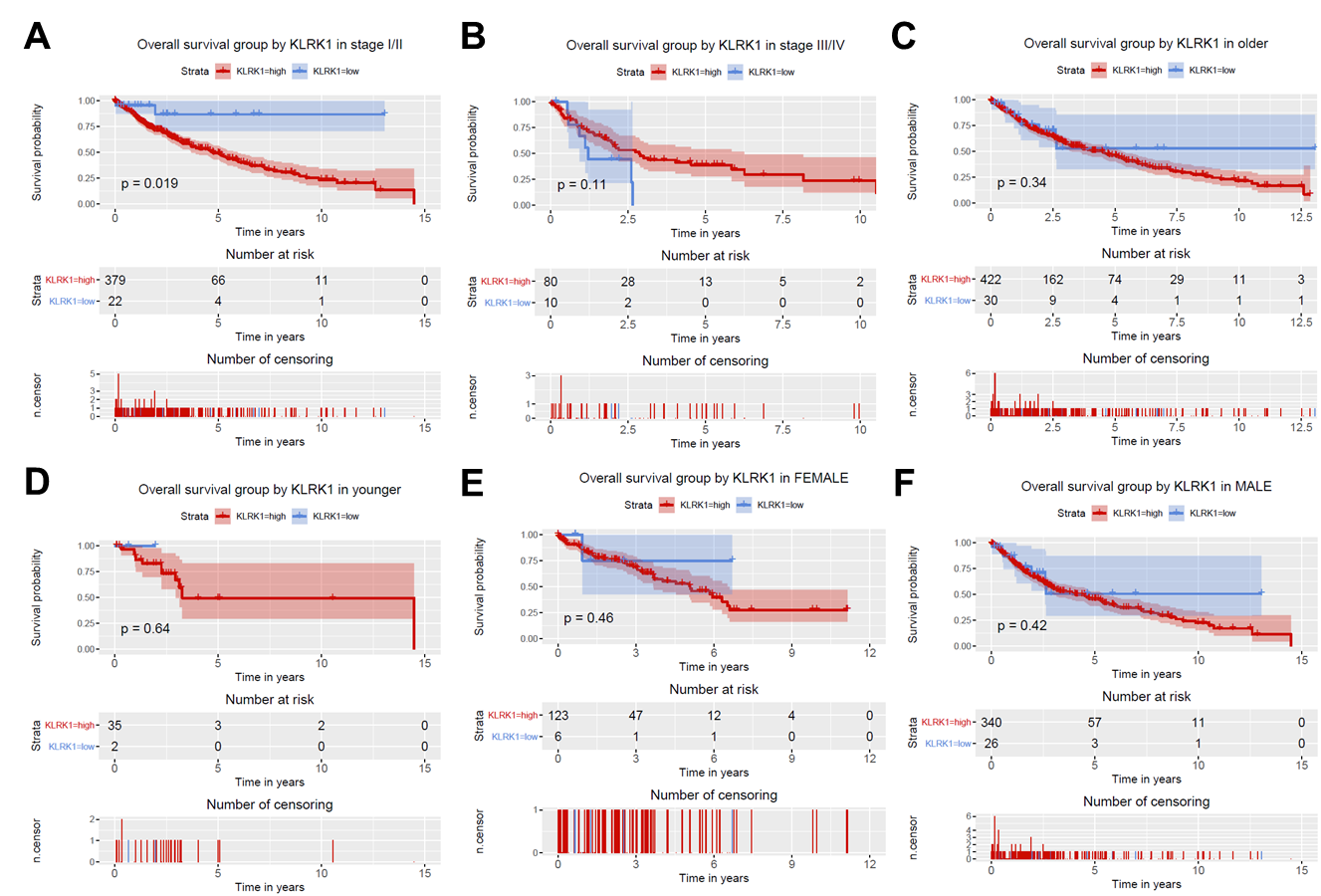


**Figure S2.** Relationship of KLRK1 expression with overall survival in all patients with lung squamous cell cancer in (A) stage I/II, (B) stage III/IV, (C) older patients, (D) younger patients, (E) female and (F) male.


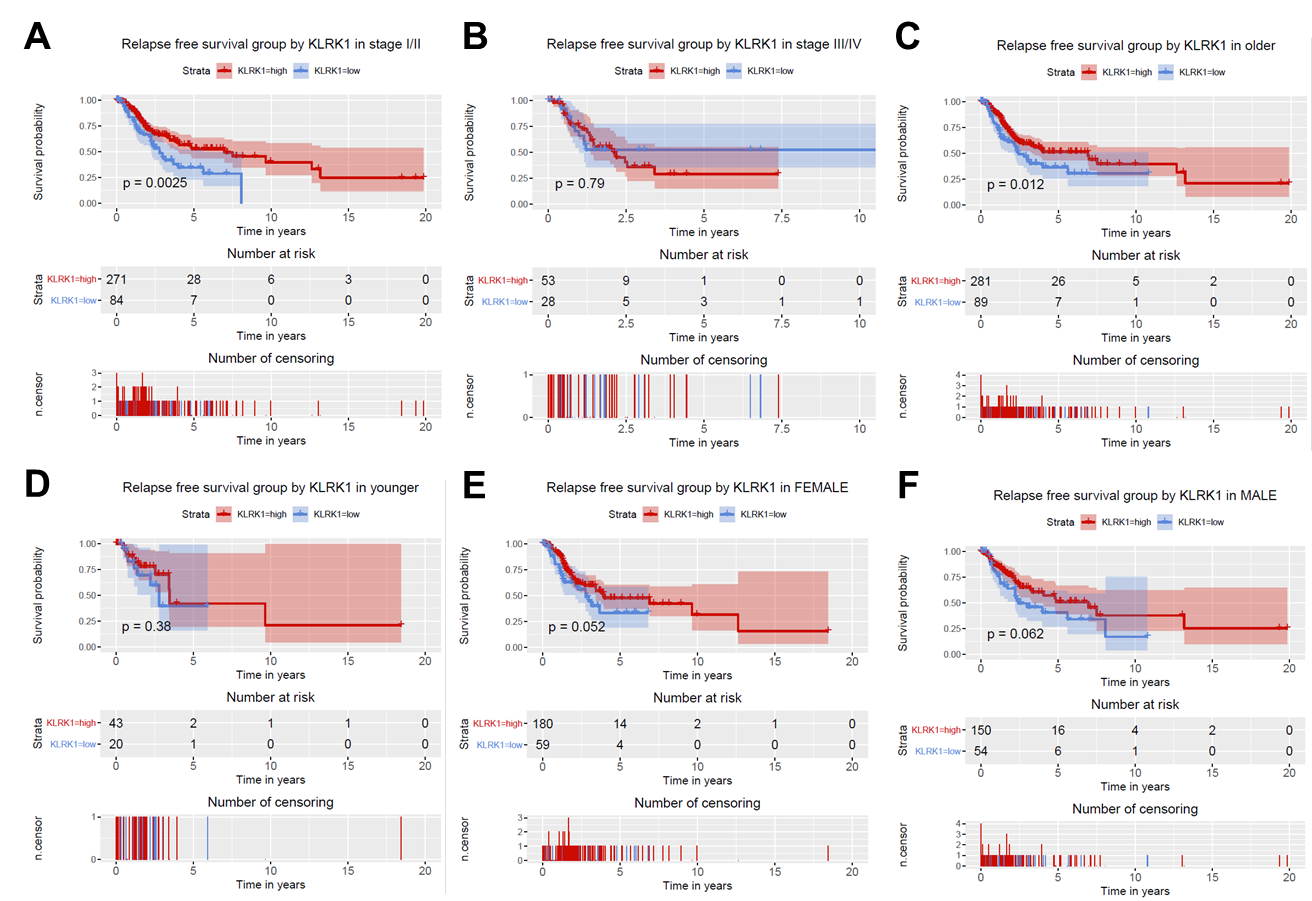


**Figure S3.** Relationship of KLRK1 expression with relapse free survival in all patients with lung adenocarcinoma cancer in (A) stage I/II, (B) stage III/IV, (C) older patients, (D) younger patients, (E) female and (F) male.


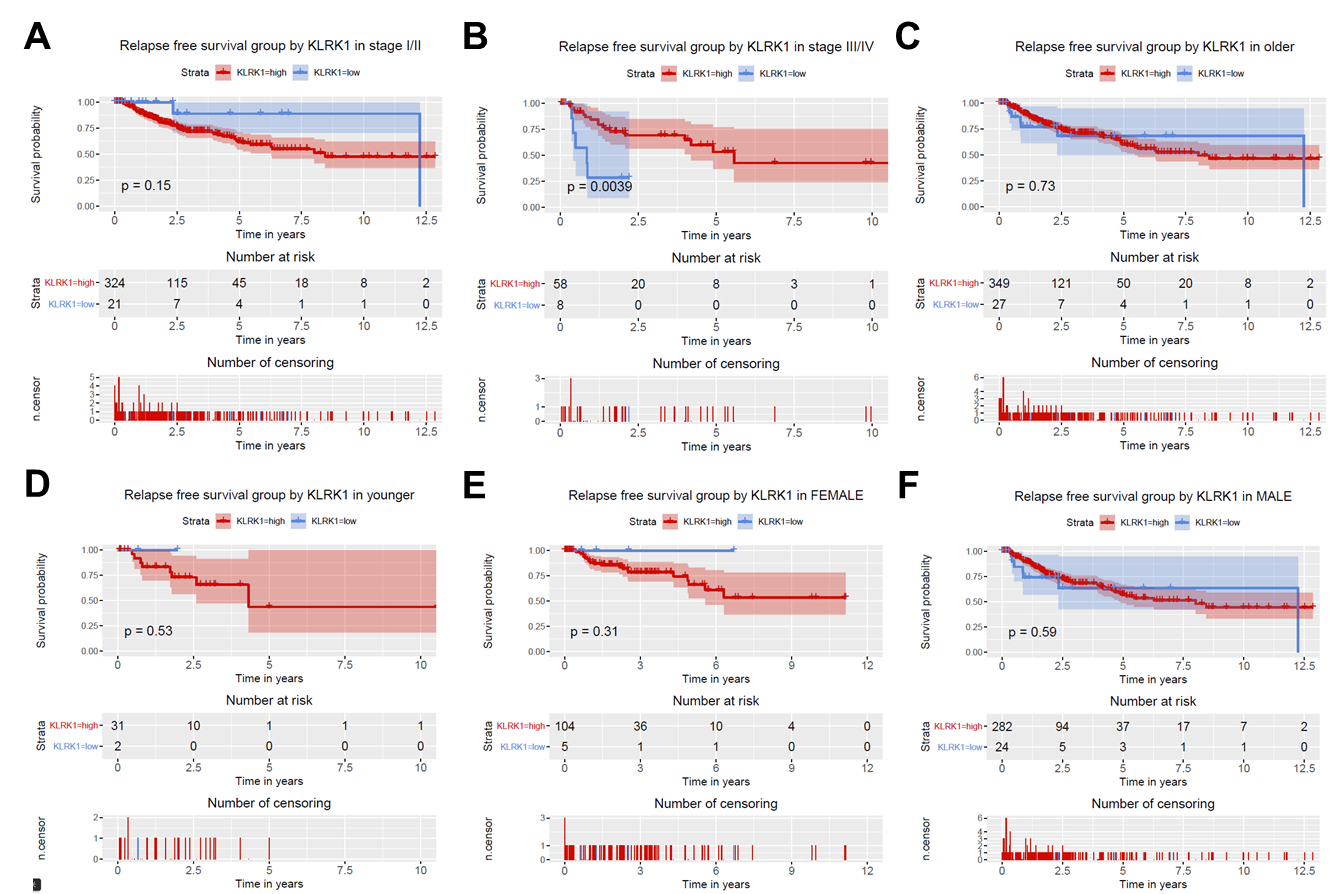


**Figure S4.** Relationship of KLRK1 expression with relapse free survival in all patients with lung squamous cell cancer in (A) stage I/II, (B) stage III/IV, (C) older patients, (D) younger patients, (E) female and (F) male.
